# Exploring 14–3-3ζ: insights into its role in cancer mechanisms and future therapeutic applications

**DOI:** 10.1186/s41065-026-00658-x

**Published:** 2026-03-03

**Authors:** Husni Farah, Munthar Kadhim-Abosaoda, Hayjaa Mohaisen-Mousa, S. Renuka Jyothi, Priya Priyadarshini-Nayak, Bethanney Janney-J, Gurjant Singh, Ashish Singh-Chauhan, Manoj Kumar-Mishra

**Affiliations:** 1 Faculty of Allied Medical Sciences, Hourani Center for Applied Scientific Research, Al-Ahliyya Amman University, Amman, Jordan; 2 College of Pharmacy, the Islamic University, Najaf, Iraq; 3 College of Pharmacy, the Islamic University of Al Diwaniyah, Munthar, Al Diwaniyah, Iraq; 4 Department of Medicinal Chemistry, Al-Turath University, Al Mansour, Baghdad, 10013 Iraq; 5 Department of Biotechnology and Genetics, School of Sciences, JAIN (Deemed to Be University), Bangalore, Karnataka India; 6 Department of Medical Oncology, IMS and SUM Hospital, Siksha ‘O’ Anusandhan (Deemed to Be University), Bhubaneswar, Odisha 751003 India; 7 Department of Biomedical, Sathyabama Institute of Science and Technology, Chennai, Tamil Nadu India; 8 Department of Physiotherapy, University Institute of Allied Health Sciences, Chandigarh University, Punjab, Chandigarh State India; 9 Division of Research and Innovation, Uttaranchal Institute of Pharmaceutical Sciences, Uttaranchal University, Dehradun, Uttarakhand India; 10 Salale University, Fitche, Ethiopia

**Keywords:** 14–3-3ζ, Tumor suppressor, Cancer progression, Signaling pathways, Chemoresistance

## Abstract

14–3-3ζ is becoming an important marker and possible treatment target because many studies link it with cancer recurrence and resistance to chemotherapy. When cancer cells make too much 14–3-3ζ, they tend to survive better, especially under stressful conditions, and they can grow even without attachment, which is usually a sign of more aggressive behavior. On the other hand, when the level of 14–3-3ζ is lowered, tumors grow slower and the cells become more sensitive to different anticancer drugs. In clinical settings high 14–3-3ζ can work as prognostic biomarker, helping to identify patients whose tumors might not respond fit to standard treatments, allowing doctors to think about more personalized therapy options. This protein also interacts with several cancer-related pathways for example working together with HER2, which can push tumors to become more invasive, and harder to treat. Many cancers show amplification of the 14–3-3ζ gene and this usually correlates with worse outcomes for patients. Even though scientists are still figuring out how directly target 14–3-3ζ, a lot of studies suggest that stopping this protein or blocking the signals it activates might make cancer cells respond better in chemo and slow the tumor from getting worse [14]. So overall, going after 14–3-3ζ looks like the pretty promising approach for dealing with therapy resistance, and it could maybe help improve patient outcomes in the future.

**Graphical Abstract:**

The 14-3-3 protein family can actually act in two totally opposite ways in cancer, which makes it kinda tricky to study. In some situations, 14-3-3 works like an anti-cancer helper because it stabilizes the p53 protein. When p53 is stable, it can stop the cell cycle and even push damaged cells into apoptosis, so those cells don’t keep dividing. But in other cases, 14-3-3 ends up doing the complete opposite and helps the tumor grow. It mainly does this by turning on the PI3K/AKT pathway, which then causes NF-κB to move into the nucleus. Once NF-κB is in the nucleus, it switches on a bunch of genes that let cancer cells survive longer, divide faster, and even migrate or invade other tissues. So overall, 14-3-3 proteins can either slow cancer down or help it get worse, depending on which signaling pathway is taking over in the cell.

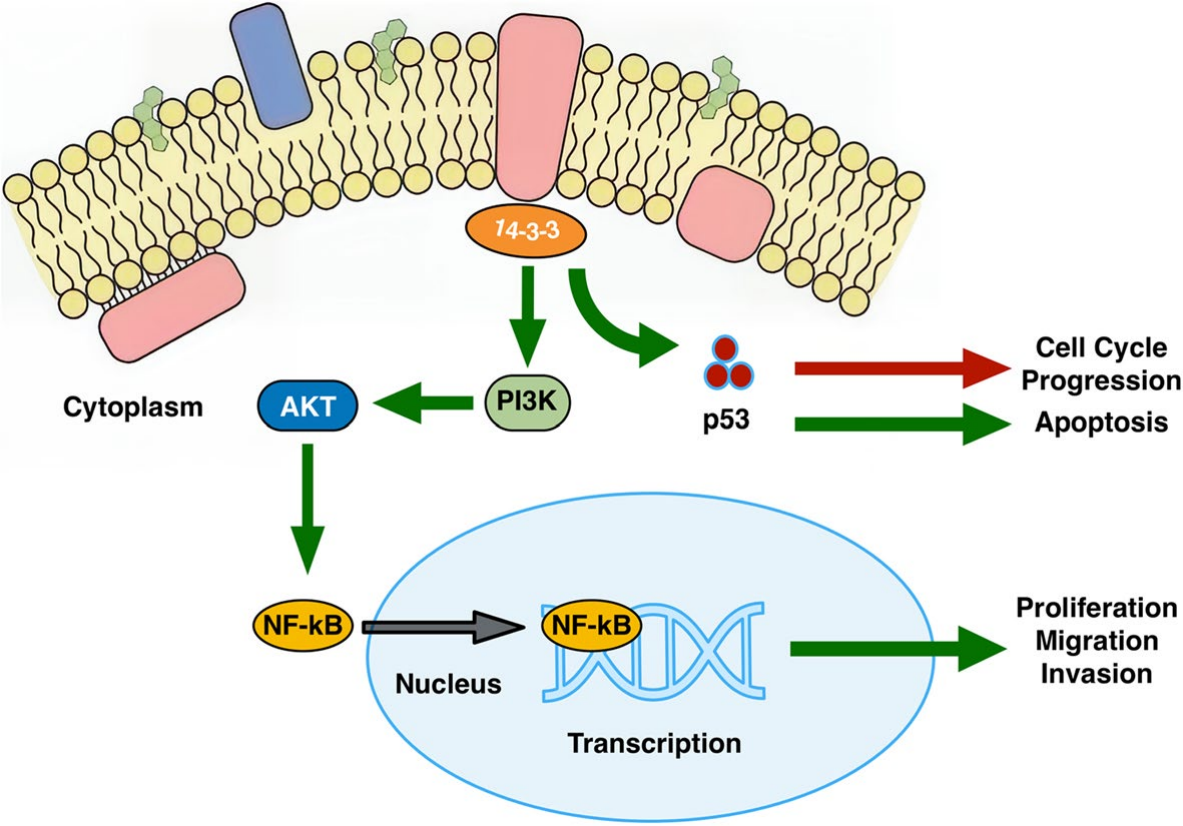

## Introduction

Cellular processes depend on on intricate coordination of frequent proteins, including membrane receptors, ion channels, and structural elements, which are vital in protective cell structure and function [1]. For cells to operate in a harmonized manner, they must possess the ability to adapt to their microenvironment and engage dynamically in protein, protein interactions [1]. Among the molecular mediators, such interactions, the 14–3-3 family of proteins has emerged as a critical regulatory component influencing the broad spectrum of cellular activities, from proliferation to apoptosis [2]. These proteins are evolutionarily conserved and ubiquitously present across eukaryotic species. In mammals, the family comprises seven isoforms: alpha/beta (α/β), eta (η), epsilon (ε), gamma (γ), sigma (σ), theta (θ), and zeta/delta (ζ/δ). The α and δ isoforms are the phosphorylated forms of β and ζ, respectively. Each isoform is encoded by the unique gene: YWHAB, YWHAG, YWHAH, YWHAE, SFN, YWHAQ, and YWHAZ [3, 4].

Structurally, 14–3-3 proteins are actually pretty small, about 30 kDa, and they usually work as dimers meaning two protein units stick together, either two of the same isoform or two different ones [5]. One of the most important parts of each monomer is the amphipathic groove, which lets the protein bind two phosphopeptides or other partner proteins at the same time [2]. Because of this, a 14–3-3 dimer can either stabilize one protein interaction or act kind of like a bridge that links two separate proteins together [6]. Their binding areas follow certain conserved patterns, known as Mode I (R-S-X-pS/pT-X-P), Mode II (R-X-F/Y-X-pS/pT-X-P), and Mode III (R-X-X-pS/pT-X-COOH), which usually show up near the C-terminal end of the target protein [7].

Each 14–3-3 polypeptide has nine α-helices that fold into a curved shape, and when two chains join, they form saddle-like dimer. The N-terminal region forms bottom of the binding pocket, while the C-terminal part shapes the walls. Some isoforms also have flexible C-terminal tail that can fold into an extra helix, which changes how easy it is for other proteins to reach the central groove [8]. The whole dimer kind of looks like a cup with a negatively charged channel in middle, about 35 Å wide and 20 Å deep [8]. Inside the groove, charged amino acids like Lys49, Arg56, Arg127, and Tyr128 help attract binding partners, and hydrophobic residues like Leu172, Val176, Leu216, Ile217, Leu220, Leu227, and Trp228, help hold them in place using van der Waals forces [9].

Some proteins including FOXO family members and AANAT, even have two binding sites for the 14–3-3 [10]. The main site binds tightly, while the second one is weaker but helps make whole complex more stable. Since 14–3-3 proteins dont have their own kinase activity, they can only bind after the target protein has already been phosphorylated [11]. Functionally, 14–3-3 proteins can keep their partner proteins in right shape, hide certain amino acids so they don’t interact with other molecules, or act as the scaffold proteins that help organize bigger protein complexes [12]. Even though many amino acids involved in dimer formation are conserved across all isoforms, small structural differences especially from which isoforms pair together create differences in which proteins they prefer for bind [11].

**Fig. 1:**
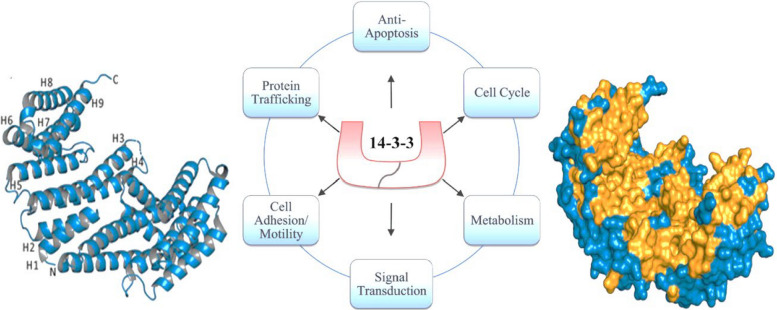
The 14–3-3 protein family has different isoforms that can pair up form either homo or heterodimers and they mainly get attention because they bind to (phosphor serine) spots on other proteins. This lets them connect with lots of different partners in the cell and help manage many pathways that keep things running normally. When these pathways get messed up, the cell can lose control and start growing in the cancer like way. Since 14–3-3 proteins sit kind of in the middle several major signaling systems that drive tumors, researchers are now considering them as possible targets for developing new cancer treatments

Another important thing is that the core of the 14–3-3 protein is pretty rigid but its C-terminal tail is very flexible. When the protein isnt bound to anything, this tail can fold into the groove and block it like self inhibiting piece. Once a ligand binds, the tail moves aside. If the tail is removed, protein actually binds targets more strongly, showing its regulatory role [13]. Because 14–3-3 proteins sit in middle of many signaling pathways, they play big roles in processes like vesicle transport, the cytoskeletal changes, cell plasticity and apoptosis. When their expression or function gets disrupted, it can contribute in diseases like cancer, neurological disorders, and metabolic problems [14]. This review mainly looks at how different 14–3-3 isoforms behave in tumors, what molecules they interact with, which pathways they regulate, and how those interactions affect on cancer biology (Fig. 1).

A unique focus is placed on the involvement of non coding RNAs, specifically microRNAs and long non-coding RNAs, in modulating the activity and expression of various 14–3-3 isoforms. Of special interest is the ζ isoform (14–3-3ζ), which has been increasingly recognized for its contribution to oncogenic transformation [15]. Higher expression of 14–3-3ζ has been linked with more aggressive cancer behavior and poorer patient outcomes in the several tumor types [16]. In this review, we focus on how 14–3-3ζ contributes to cancer development and why it may work as both a biomarker and a possible therapeutic target. This viewpoint adds new angle for cancer research by suggesting that 14–3-3ζ could be directly targeted in future treatments. As precision medicine keeps advancing, understanding the exact mechanisms of 14–3-3ζ becomes even more important. By showing the strong link between 14–3-3ζ and tumor progression, we highlight its value as an important direction for upcoming studies.

## 14–3-3 Proteins in human malignancies

### Mutations and functional alterations of 14–3-3 proteins

Even though actual somatic mutations in 14–3-3 genes dont happen very often in the human cancers, the research we do have show that even small changes in their structure or function might still affect how tumors form or behave. For example, one breast cancer study found that patients with higher mutation rates across different 14–3-3 isoforms tended to have the worse clinical outcomes, even though the study didnt fully explain what kind of mutations were involved [17]. Also in many epithelial cancers, some 14–3-3 proteins especially 14–3-3σ are shut down through the epigenetic mechanisms. This kind of silencing acts almost the same as a loss-of-function mutation, since the protein basically disappears from cells [18].

Experiments that introduce specific mutations 14–3-3 proteins also help show how structural changes can influence their behavior in cancer. For example phosphomimetic mutants like S58E or S58D in 14–3-3ζ, are often used to mimic phosphorylation, but they dont accurately copy what really happens at Ser58. Instead, they cause the protein to shift more toward monomer form which changes how stable it is and where it goes inside cancer cells [19]. Another case is the S60A mutation in 14–3-3β, which stops protein from interacting with β1 integrin but surprisingly doesn’t stop cells from spreading. By contrast, mutations in the amphipathic groove like the LF mutant block cell migration and spreading altogether, even though integrin binding stays normal [20]. This shows that different structural regions of 14–3-3 protein control different cell behaviors, and when these regions are altered, cancer related processes can be affected.

Even though we still dont have enough data on real tumor associated mutations in 14–3-3 genes, the fact that some isoforms get silenced so often in the epithelial cancers, and that specific structural domains have important roles suggests that changes in 14–3-3 structure or expression could help push cancer progression [18]. There is a real need for larger genomic studies to figure out how common these mutations actually are or how they affect patients, and whether they could serve as biomarkers. Understanding these alterations better may help reveal key pathways that support malignant transformation and points toward new therapeutic strategies.

### Amplification of the 14–3-3ζ Gene in tumorigenesis

One the major molecular change that helps drive cancer development is the amplification of certain genomic regions, which increases the number of gene copies and often boosts their expression [21]. When this happens, tumor cells may gain survival or growth benefits, and the amplified genes can sometimes be used to predict disease severity or even serve as drug targets [22]. The gene for 14–3-3ζ, called YWHAZ, is located on chromosome 8q22.3, a region that is commonly amplified in cancers like breast cancer and others. Many studies show that when YWHAZ is amplified, tumors usually have higher levels of the 14–3-3ζ protein as well [23]. For example, array-based CGH and expression profiling in urothelial carcinomas found that advanced tumors often had amplification of the region containing YWHAZ. Among the genes there, YWHAZ and POLR2K showed the strongest link between copy number increases and higher expression [24]. In head and neck squamous cell carcinoma (HNSCC), tissue microarray studies reported YWHAZ amplification, often due to chromosome 8 polysomy, in about 30–40% of cases [3]. Likewise, 14–3-3ζ protein overexpression appeared in roughly 77% of HNSCC samples, and many tumors showed both gene amplification and protein overexpression [25].

Similar findings have been reported in breast cancer, where chromosome 8 polysomy also raises YWHAZ copy numbers [4]. FISH studies showed that YWHAZ amplification closely matched increased 14–3-3ζ protein levels by immunohistochemistry [25, 26]. Another study identified a group of 12 genes, including YWHAZ, in the amplified 8q22 region that were linked to metastatic recurrence [5]. Amplification of this region increased expression of these genes and acted as an independent marker for predicting relapse in breast cancer [26]. Together, these results suggest that gene amplification is a key reason why 14–3-3ζ is overexpressed in many tumors [27]. However, other regulatory factors, like changes in transcription, translation, or protein stability, may also raise 14–3-3ζ levels. Detecting YWHAZ amplification could help identify patients at higher risk of recurrence [28, 29]. Understanding these regulatory mechanisms is important for creating new therapies, and more research is still needed to see how 14–3-3ζ changes influence cancer behavior and patient outcomes.

### Oncogenic functions of 14–3-3 subtypes

14–3-3 proteins influence tumor development by regulating cell growth and survival. They interact with the TSC1/TSC2 complex (a tumor-suppressive pathway) to modulate the cell cycle [30, 31]. Earlier studies have identified four 14–3-3 subtypes (γ, ε, ζ, and σ) are upregulated by TSC1/TSC2 [32]. Further work showed that phosphorylation-dependent binding of TSC2 to 14–3-3 inhibits TSC2 function, suggesting 14–3-3 promotes tumorigenesis through this interaction [33]. In hematopoietic progenitor cells, 14–3-3 expression increases under IL-3 stimulation [34]. Overexpression of 14–3-3 in Ba/F3 cells eliminated IL-3 dependence and activated PI3K/Akt and MAPK pathways, indicating a role for 14–3-3 in promoting cell growth and survival in normal progenitor cells [35]. In lung adenocarcinoma A549 cells, 14–3-3 overexpression enhanced proliferation and induced more invasive morphology [36], suggesting a role in malignant phenotypes. 14–3-3 proteins also regulate apoptosis by interacting with Bax and Bad [37]. During DNA damage, 14–3-3 deficiency causes Bax translocation to mitochondria, triggering rapid apoptosis [38]. With 14–3-3 present, phosphorylation-dependent binding sequesters Bax in the cytoplasm and inhibits apoptosis [39]. Since Bad suppresses the anti-apoptotic function of Bcl-2 and Bcl-xL, 14–3-3 regulation of Bad also controls apoptosis [40]. Reduced 14–3-3 expression upregulates stress-induced apoptosis and sensitivity to JNK/p38 pathways [41], while 14–3-3 overexpression enhances cell adhesion and contact. 14–3-3 proteins may also influence tumor signaling through RIN1 [42]. A newer splice form of RIN1 that contains both Ras-binding and 14–3-3-binding regions was recently found in colon cancer cells [43]. This version is highly expressed in both gastric and colon cancer lines, stays mainly in the cytoplasm, and can bind to 14–3-3 proteins, which means it might take part in signaling pathways that help tumor growth [43]. Figuring out how each 14–3-3 subtype behaves on its own could give important clues about different steps in cancer biology. Because these interactions are quite complicated, more studies are needed to understand how they might be used for therapy in the future.

#### 14–3-3σ Subtype

The 14–3-3σ subtype is one of the most studied isoforms, and it plays a noticeable role in tumor development, therapy resistance, and recurrence of disease (14). Immunohistochemistry on 50 colorectal cancer samples showed that 14–3-3σ was much more common in tumor tissue (58%) compared to normal colon mucosa [6]. Its expression was associated with age, tumor size, and lymph node metastasis, although it did not show big differences with gender, tumor grade, or serosal invasion [44]. In pancreatic tumors, 14–3-3σ was positive in 70% of intraductal papillary–mucinous tumors and in all invasive ductal carcinomas [45], hinting that it may be involved early in pancreatic cancer development.

Even though people often talk about this isoform in cancer, 14–3-3σ mainly acts like a tumor suppressor [46, 47]. It is often silenced by CpG promoter methylation, which is a critical step during the formation of several cancers [48, 49]. Treating colorectal cancer cell lines with 5-azacytidine increased promoter methylation of 14–3-3σ and other genes [50]. In melanoma lines and melanocytes, the 14–3-3σ promoter was also hypermethylated, turning off its expression and helping melanoma progression [51]. Nasopharyngeal cancer shows a similar pattern: methylation occurred in four tested cancer lines, but not in the normal epithelial cells. When cells were treated to remove methylation, 14–3-3σ expression went up again, and tumors displayed much higher methylation (84%) than nearby normal tissue (28%) [52].

14–3-3σ may also have value as a diagnostic marker. When combined with mesothelin in pancreatic cancer samples, detection accuracy reached about 90%, and expression was almost exclusive to cancer tissues [53]. In pancreatic intraepithelial neoplasia, 14–3-3σ increased stepwise with worse lesion severity [54]. ELISA tests of serum and pancreatic fluid also suggested that this isoform might serve as a biomarker for pancreatic cancer [55].

#### 14–3-3ζ Subtype

The 14–3-3ζ protein is involved in many cancer-related processes across different tumor types. In many cancers, high levels of 14–3-3ζ are strongly linked with tumor growth and overall progression of the disease [56]. For example, in glioblastoma, patients who have more 14–3-3ζ usually show more advanced tumor stages, more recurrence, and shorter survival times [57]. Experiments in the lab also show that when this protein is overexpressed, glioblastoma cells grow, migrate, and invade faster, mostly because the PI3K/AKT pathway gets activated [58]. When researchers silence 14–3-3ζ, these aggressive behaviors slow down, and even the mitochondrial function and cell metabolism start to change, meaning this protein probably has wider roles than just signaling [12]. In gliomas, 14–3-3ζ also interacts with hypoxia related factors, working with HIF 1α and VEGF during low oxygen conditions [59]. It can also activate the ERK pathway through Raf, boosting survival pathways; When 14–3-3ζ is blocked, survival genes drop and cell migration is reduced in glioblastoma models [58].

14–3-3ζ is also highly studied in gastrointestinal cancers. In oral squamous cell carcinoma and tongue cancer, its expression is much higher and is linked to more aggressive tumor behavior and worse clinical outcomes, partly through pathways like FOXO3a [60]. In oral cancers, 14–3-3ζ levels rise step by step from normal mucosa, to precancerous lesions (69%), and then to full tumors (79%) [61]. This rise starts early, during hyperplasia, and increases with more severe dysplasia. The protein can bind NF-κB, β-catenin, and Bcl-2, which suggests it pushes tumor growth by activating several cancer-related signaling routes [61].

In esophageal squamous cell carcinoma, almost all tumors, (95%) showed cytoplasmic staining of 14–3-3ζ and 63% also showed nuclear staining, while normal tissue barely expressed it [62]. These changes were connected to abnormal NF-κB and S1P signaling, and researchers also found that 14–3-3ζ works in opposition to S1PR2 in this cancer [63]. Similar patterns happen in the esophagogastric junction cancers, where high 14–3-3ζ is linked with lymph node involvement and higher recurrence rates [64]. In gastric cancer, 14–3-3ζ affects apoptosis by binding Bax; specific phosphorylation steps can change this interaction and push Bax into mitochondria, leading to cell death [7]. High expression of 14–3-3ζ is also related to worse clinical features and is negatively associated with miR-375 levels [65]. Since miR-375 binds directly to the 3′ UTR of 14–3-3ζ, raising miR-375 lowers 14–3-3ζ and triggers apoptosis. In cholangiocarcinoma, higher 14–3-3ζ in advanced stages is tied to tumor aggressiveness, and knocking it down weakens metastatic traits [66, 67]. In hepatocellular carcinoma (HCC), autoantibodies against 14–3-3ζ indicate it could be the early biomarker. The protein forms a complex with αB Crystallin that promotes EMT and therapy resistance through the ERK/Fra-1/Slug pathway [68, 69].

**Fig. 2:**
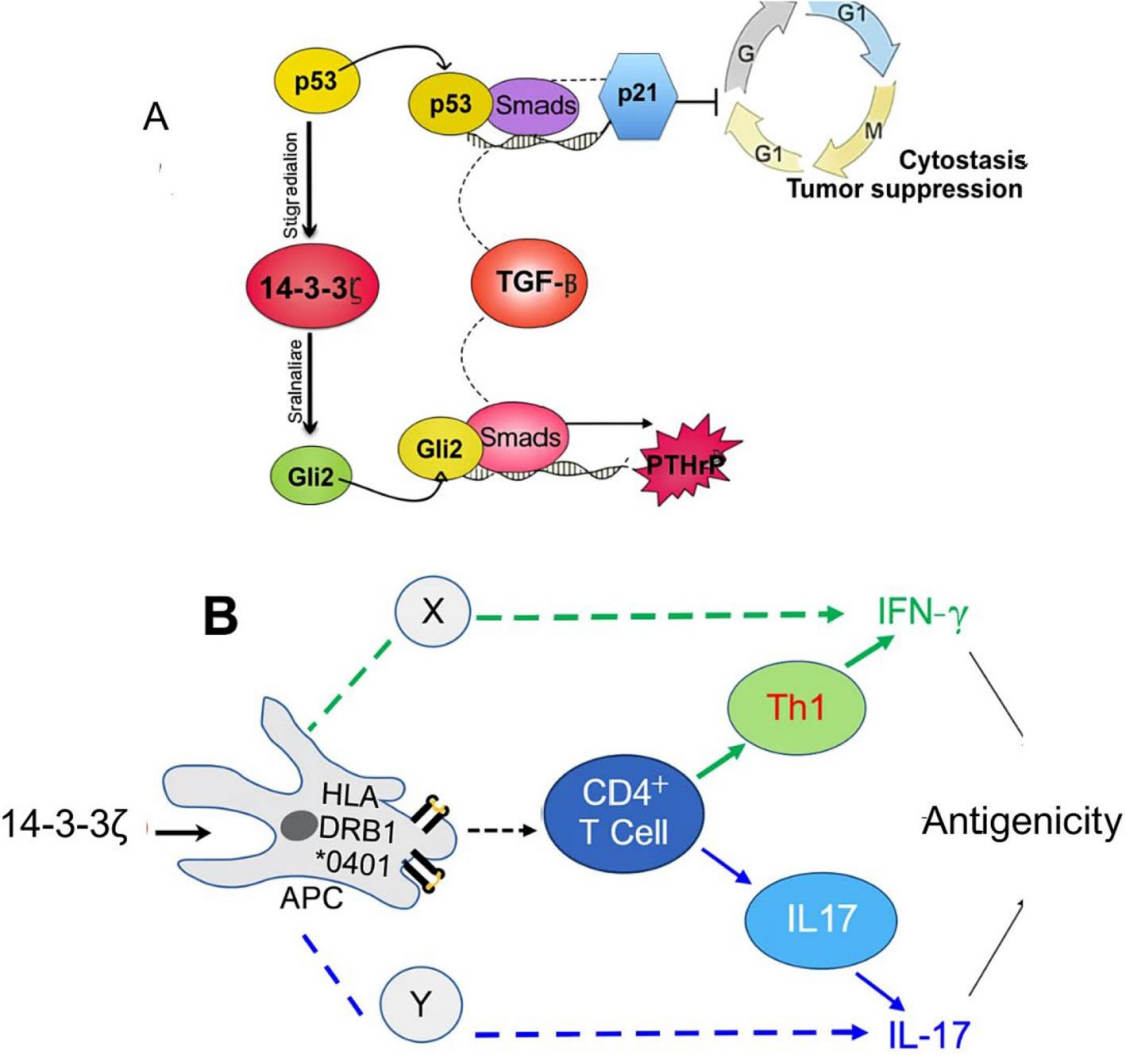
**A** The 14–3-3ζ-mediated alteration in Smad binding partners, shifting from p53 to Gli2, could function as both biomarkers and potential therapeutic targets in the context of TGF-β-driven cancer progression. **B** 14–3-3ζ induces antigenic responses by increasing IFN-γ and IL-17 production in human peripheral blood mononuclear cells (PBMCs) through both MHC class II-dependent and independent mechanisms, respectively. This effect influences CD4 + T cell polarization as well as other cytokine-producing cell types. The impact of 14–3-3ζ on Th1 cells mainly relies on MHC class II involvement, while non-CD4 + cells producing IFN-γ and IL-17 show greater sensitivity to 14–3-3 inhibition

Head and neck squamous cell carcinoma also shows strong 14–3-3ζ expression, which promotes EMT and reduces senescence [8]. Silencing the protein changes the cell-cycle pattern and increases apoptosis markers (20). In lung cancers such as NSCLC, 14–3-3ζ is again overexpressed and linked with advanced disease and worse survival. Its knockdown increases sensitivity to cisplatin and reduces tumor size in animal models [70, 71]. High expression in NSCLC is associated with stage, poor differentiation, and lower survival rates [72]. Bladder cancer shows YWHAZ gene amplification in later stages, and high 14–3-3ζ correlates with metastasis and poor prognosis [9]. It also interferes with pro-apoptotic genes and supports chemoresistance [73]. In prostate cancer, androgen signaling regulates 14–3-3ζ, increasing cell survival and mobility through androgen receptor interaction and pathways like JAK2/STAT3 and HO-1 [58]. In castration-resistant disease, elevated 14–3-3ζ is linked with poorer outcomes and enhanced migration via the RAC-PAK pathway [10]. Lowering 14–3-3ζ boosts radiation-induced apoptosis through p38 and JNK activation [74]. In leukemia, both drug-sensitive and resistant AML cells express 14–3-3ζ, and its knockdown makes them more drug-sensitive by lowering anti-apoptotic signals. Loss of 14–3-3ζ also increases anoikis, showing its role in helping cells survive when detached [75, 76]. The role of 14–3-3ζ extends into female reproductive cancers as well. In ovarian cancer, its expression varies by subtype and clinical stage, making it a possible biomarker [77]. Ovarian tissues frequently overexpress this subtype, and silencing it decreases tumorigenicity and shifts metabolism, mainly through PI3K/AKT pathway downregulation [77]. In breast cancer, particularly ductal subtypes, its expression aligns with poor prognosis and increased recurrence [11]. 14–3-3ζ enhances PI3K signaling through interaction with p85, and collaborates with ErbB2 to facilitate invasiveness and EMT through TGFβ/Smad activation [78, 79] (Fig. 2).

#### Other 14–3-3 Subtypes

Studies have confirmed the negative regulatory effect of Sulindac, a non-steroidal anti-inflammatory drug, on 14–3-3ε expression in colorectal cancer cells (HT-29 and DLD-1) in a time- and dose-dependent manner [38, 80]. This effect occurred through PPARδ pathway inhibition, as PPARδ overexpression reversed 14–3-3ε suppression [12]. Sulindac also decreased cytoplasmic Bad levels, while stable 14–3-3ε expression prevented apoptosis. Selective COX-2 inhibitors similarly suppressed 14–3-3ε in HT-29 cells [80, 81]. These findings suggest a new mechanism for non-steroidal anti-inflammatory drugs to induce colorectal cancer cell death via the PPARδ/14–3-3ε pathway [82]. Curcumin (10–80 mmol/L) further reduced PPARδ, 14–3-3ε, and VEGF expression in HT-29 cells, triggering apoptosis and caspase-3 activation [83]. This supports 14–3-3ε's role in colorectal cancer cell survival [84].

14–3-3γ has been found at much higher levels in hepatocellular carcinoma tissues, something confirmed by Western blot tests [33]. In lung cancer H322 cells, when researchers forced the cells to overexpress 14–3-3γ, the cells started showing abnormal DNA replication and became polyploid, meaning they had extra sets of chromosomes [85, 86]. These cells also became resistant to microtubule-targeting drugs and could jump back into the cell cycle even without going through normal mitosis. This basically suggests that 14–3-3γ helps cancer cells skip important mitotic checkpoints, which increases genomic instability and might push tumor development forward [85]. In gastric cells infected with *H. pylori* (AGS cells), 14–3-3τ levels also went up, along with nine other proteins related to cell growth, adhesion, and cancer-linked pathways. This pattern hints that 14–3-3τ could play a part in *H. pylori*-related stomach disorders and maybe even gastric cancer formation [87].

## 14–3-3 Proteins as Biomarkers for cancer diagnosis, prognosis, and treatment resistance

Research so far shows strong links between changes in 14–3-3 protein levels and clinical outcomes in cancer patients. For example, high expression of 14–3-3ζ (YWHAZ) is usually connected with worse prognosis in many types of cancer, with hazard ratios ranging from 2.3 up to 6.2 in multivariate analyses [23, 88, 89]. Basically, patients who have higher amounts of this isoform tend to have poorer survival rates, which makes 14–3-3ζ a meaningful prognostic biomarker. Meanwhile, the 14–3-3σ isoform seems more helpful for diagnostic purposes. It can help differentiate between some cancer types, and studies show strong odds ratios for identifying breast cancer or telling prostate cancer apart from bladder cancer [90, 91].

Even though the results shown in Table 1 look statistically significant, the numbers vary a lot between studies, and some of the research used pretty small groups of patients. Because of this, we need to be cautious when interpreting the findings. Larger, multi-center studies are still necessary to verify how reliable these markers really are and to understand better why different 14–3-3 isoforms behave in different ways across cancer types. Overall, these observations highlight why 14–3-3 proteins are becoming more important in oncology and why we need more research to figure out their biological roles and future therapeutic value (Table 1).

**Table 1:** Quantitative clinical significance of 14–3-3 proteins in cancer

14–3-3 ISOFORM	CANCER	CLINICAL SIGNIFICANCE	VALUE	HAZARD/ODDS RATIO	P-VALUE	REF
ζ	EGJA	Prognosis	4.49	1.74–13.06	0.002	[13]
ζ	DLBCL	Prognosis	6.21	3.21–12.02	< 0.05	[14]
ζ	Gastric	Prognosis	2.30	1.00–5.03	0.049	[15]
σ	Breast	Diagnosis	302	−	< 0.05	[16]
σ	Prostate vs. Bladder	Differentiation	0.03	0.00–0.22	0.001	[17]
σ	S-GCT vs. E-GCT	Differentiation	0.06	0.01–0.50	0.009	[18]
σ	ccRCC vs. pRCC	Differentiation	0.05	0.01–0.26	< 0.001	[19]
η	Lung	Prognosis	3.45	1.50–8.00	0.015	[20]
ε	Colorectal	Prognosis	5.00	2.00–12.50	0.008	[21]
ζ	Ovarian	Prognosis	2.10	1.05–4.20	0.035	[22]
σ	Liver	Diagnosis	150	−	< 0.01	[23]
σ	Pancreatic	Prognosis	1.80	1.20–2.70	0.041	[24]

### 14–3-3 Protein as the diagnostic and prognostic biomarkers

Finding new biomarkers that can help predict cancer recurrence or chance of metastasis is really important, because it allows doctors to intervene earlier with stronger or more targeted treatments. Being able for identify markers linked to therapy resistance is also valuable, since it could help clinicians choose the treatments that are more likely to work and avoid giving patients drugs that might not help, which also reduces unnecessary side effects [92]. Higher levels of 14–3-3ζ have been reported in many different cancers, which suggests that this protein might take part in several key pathways that support tumor growth and spread [93]. Even though we are still learning about the exact clinical role of 14–3-3ζ in human cancers, newer studies are already showing a strong association between its expression and patient outcomes, meaning it could become an important marker for prognosis in the future [94].

Recent studies increasingly point to the potential role of 14–3-3ζ as a prognostic biomarker, capable of indicating the risk of recurrence and resistance to standard cancer treatments [95]. In head and neck or oral squamous cell carcinoma (HNOSCC), patients with high 14–3-3ζ levels experienced shorter disease-free intervals than those with lower levels, although the difference in median disease-free survival was not statistically significant. [25]. However, in cases where both 14–3-3σ and 14–3-3ζ were overexpressed, patients had a markedly shorter disease-free interval [96]. A similar pattern has been observed in NSCLC, where overexpression of 14–3-3ζ was significantly correlated with poorer survival outcomes and more frequent disease relapse. High levels of 14–3-3ζ also showed a strong association with tumor grade and clinical staging. Importantly, in this NSCLC cohort, 14–3-3ζ overexpression emerged as the sole independent variable predicting both recurrence and reduced survival [97].

Parallel results were also seen in breast cancer, where tumors in more advanced stages that showed high 14–3-3ζ levels were much more likely to come back and were linked with worse outcomes overall [26]. This protein also seemed match up with ErbB2 overexpression and higher tumor grades. Even after considering strong prognostic markers like lymph node status or ErbB2, 14–3-3ζ still acted as its own independent sign of recurrence risk [27, 94]. Patients who had combination of high ErbB2, positive lymph nodes, and high 14–3-3ζ expression had an especially high chance of developing distant metastasis [27]. Supporting this idea, breast tumors that overexpressed both 14–3-3ζ and ErbB2 show noticeably higher recurrence rates and a stronger tendency to spread [98]. Overall, these findings imply that checking 14–3-3ζ levels in tumors might be pretty useful way for predict recurrence and disease worsening in many types of cancer [95].

In summary, 14–3-3ζ seems to be a very promising diagnostic and prognostic biomarker in cancer. Its increased expression across different malignancies suggests it plays an important role in pathways that push tumor progression, which makes it helpful for predicting recurrence and metastasis. Being able to identify consistent biomarkers like 14–3-3ζ is crucial for early treatment planning and avoiding therapies that may not be effective. Newer studies show a clear link between 14–3-3ζ expression and clinical outcomes. For example, in head and neck or oral squamous cancers, high levels are related to shorter disease-free intervals, and in non-small cell lung cancer (NSCLC), overexpression is closely tied to worse survival and more frequent relapse. Likewise, in breast cancer, increased 14–3-3ζ is associated with more advanced tumors and ErbB2 overexpression, making it a strong independent predictor of recurrence. Together, these observations support idea that measuring 14–3-3ζ could be a valuable tool for forecasting recurrence and disease progression across many cancer types, improving treatment planning and patient outcomes. Overall, 14–3-3 proteins including 14–3-3ζ show strong potential for cancer diagnosis and prognosis. Their expression patterns array with tumor behavior and patient survival. Because of this, they may help guide more personalized treatment choices based on a tumors molecular profile. Still, more clinical research is needed for fully confirm and expand their use in practice.

### 14–3-3ζ and resistance to cancer therapies

Chemotherapy and radiotherapy remain the main treatment choices for many cancers today [99]. However, a major challenge is that patients often respond very differently, and tumors may slowly become resistant, or they can already be resistant from the beginning. This resistance, particularly when cancer cells successfully escape apoptosis, is a key reason why these treatments do not work well [100]. Growing evidence suggests that overexpression of 14–3-3ζ is closely connected to this problem, mainly because the protein strongly promotes cell-survival signaling and suppresses apoptosis, making tumor cells harder to eliminate [101].

Studies indicate that when 14–3-3ζ expression is high, cancer cells usually react less effectively to many chemotherapeutic agents. For example, patients receiving tamoxifen for hormone-dependent tumors or anthracycline drugs such as doxorubicin and epirubicin often have poorer outcomes when their cancers show increased 14–3-3ζ levels [102]. These findings support the idea that 14–3-3ζ is an important driver of chemoresistance and could also serve as a biomarker to predict which patients are unlikely to benefit from standard cytotoxic therapies [38]. Therefore, measuring 14–3-3ζ expression before starting treatment might help clinicians design more suitable, individualized therapy plans for each person [38].

This link is even more obvious in breast cancer. Tamoxifen therapy has been reported to increase 14–3-3ζ expression, which may partly explain why some tumors gradually become resistant to this drug over time [27]. This rise appears to be triggered through activation of the estrogen receptor (ER) in breast cancer cells, even though the elevated 14–3-3ζ levels do not seem to be directly regulated by estrogen itself [103]. Two genes strongly induced by tamoxifen, YWHAZ (which encodes 14–3-3ζ) and LOC441453, were found to be much more highly expressed in ER-positive patients who later had their cancer recur after tamoxifen therapy [104]. These observations point to a possible mechanism where excess 14–3-3ζ helps drive tamoxifen resistance, making treatment less effective over time [105].

Moreover, genomic amplification of the 8q22 chromosomal region has been implicated in the enhanced expression of a subset of 12 genes located within this region, which is linked to chemotherapeutic resistance in breast cancer [106]. Overexpression of these genes in tumor tissues has been associated with a poor clinical prognosis in untreated patients and a higher likelihood of cancer recurrence even after receiving adjuvant chemotherapy [26, 107]. Functional screening using siRNA targeting this gene cluster identified that downregulating YWHAZ and LAPTM4B led to increased sensitivity of breast cancer cells to certain chemotherapeutic agents, particularly anthracyclines, while having no significant effect on the response to cisplatin or paclitaxel [108]. Elevated levels of YWHAZ or LAPTM4B expression in tumor samples have been associated with less favorable outcomes following chemotherapy [28]. Furthermore, increased expression of either or both of these genes in patient tumors correlated with reduced clinical responsiveness to the anthracycline drug epirubicin, but not to other agents such as cisplatin or docetaxel [26, 109].

In the case of diffuse large B-cell lymphoma (DLBCL), elevated 14–3-3ζ expression was identified in cell lines that demonstrated resistance to CHOP, a standard combination therapy comprising cyclophosphamide, doxorubicin, vincristine, and prednisone [110]. Experiments showed that when researchers knocked down 14–3-3ζ in drug-resistant DLBCL cell lines, the cells became sensitive again to CHOP-induced apoptosis [110]. In patients with DLBCL, higher 14–3-3ζ levels were also detected, which supports the idea that this protein might play an important part in clinical resistance to CHOP therapy. A similar pattern was seen in prostate cancer cells resistant to the camptothecin analog 9NC6, where 14–3-3ζ levels were noticeably higher [88, 111]. Other 14–3-3 isoforms have also been linked to resistance based on different in vitro studies and clinical samples [112]. Altogether, these results suggest that combining FISH testing for YWHAZ with protein expression analysis of 14–3-3ζ could help predict both how aggressive the cancer might be and how well a patient might respond to treatment [113]. Knowing a tumor’s 14–3-3ζ status could guide more personalized treatment choices.

General 14–3-3ζ is clearly involved in resistance to several cancer therapies, including the chemotherapy and radiotherapy, which are still the main treatments for many cancers. Differences in how patients respond and how tumors develop resistance make treatment outcomes difficult. Because 14–3-3ζ prevents apoptosis and supports survival pathways, its overexpression is strongly linked to chemoresistance. High levels of the protein are associated with weaker responses to tamoxifen, anthracyclines, and other common drugs, which is why it is being looked at as a potential marker for predicting treatment success. For example in the breast cancer, tamoxifen can increase 14–3-3ζ levels through ER-related pathways, possibly leading to resistance. Amplification of YWHAZ, the gene for 14–3-3ζ, is also tied to recurrence risk and poor prognosis. In cancers like DLBCL and prostate cancer, high 14–3-3ζ expression matches with clear resistance to standard therapies, which makes this protein a promising target for overcoming these obstacles. Together, these findings support checking 14–3-3ζ levels in tumors to tailor treatment strategies and hopefully improve patient outcomes. Understanding how 14–3-3ζ drives treatment resistance can also help in designing combination therapies that may work better in difficult cases. This makes it even more important to investigate therapeutic approaches aimed at blocking 14–3-3ζ-related pathways.

## Interactions between non-coding RNAs and 14–3-3

### MicroRNAs

MicroRNAs (miRNAs) are really important regulators of YWHAZ, the gene that codes for 14–3-3ζ, in different cancers. Several studies show that certain tumor-suppressor miRNAs can directly bind to the 3′ UTR of YWHAZ mRNA, which lowers the amount of 14–3-3ζ protein the cell makes [15]. But in most cancers these miRNAs are downregulated, so 14–3-3ζ levels go up and help the tumor progress [29]. When these miRNAs suppress 14–3-3ζ, they block major cancer-related behaviors like proliferation, migration, invasion, and even therapy resistance [15]. Restoring the miRNA usually reverses the cancer traits, but if 14–3-3ζ is overexpressed again, the tumor behavior comes back, showing how important this regulatory relationship is [30]. Many experiments, like luciferase assays and Western blots, confirm the inverse link between these miRNAs and 14–3-3ζ across different cancer types [114]. This miRNA-14–3-3ζ system also affects several major signaling pathways, including PI3K/AKT [115, 116], Wnt/β catenin [117], and p38MAPK/ERK [118], all of which control survival, metastasis, and drug response. Because of these, targeting miRNAs that regulate 14–3-3ζ might become the good therapeutic strategy for dealing with drug resistance and aggressive tumor behavior. Table 2 gives an overview of the main miRNA–14–3-3ζ interactions reported in cancer.

**Table 2:** miRNA-14–3-3ζ (YWHAZ) interactions in cancer

miRNA	Cancer	Expression		Key Effects	Ref
		miRNA	YWHAZ		
miR-3074-5p	NSCLC	↓	↑	↑ Chemosensitivity ↓ Proliferation ↓ Migration	[119]
miR-30c	Breast Pancreatic	↓	↑	↓ GEM-DOX resistance ↓ P-gp	[118, 120]
miR-1-3p	CRC	↓	↑	↓ Proliferation ↓ Invasion ↓ EMT	[121]
miR-193a	Gastric	↓	↑	↑ Survival ↓ Invasion ↓ Migration	[122]
miR-1225-5p	Osteosarcoma	↓	↑	↑ G1 arrest ↓ Proliferation ↓ Metastasis	[123]
miR-31-5p	Prostate	↓	↑	↑ Apoptosis (via PI3K/Akt) ↓ Proliferation	[115]
miR-204-5p	ESCC	↓	↑	↑ Apoptosis ↓ Proliferation ↓ Metastasis	[116]
miR-802	Ovarian	↓	↑	↑ Apoptosis ↓ Proliferation ↓ Metastasis	[124]
miR-375	Gastric Renal	↓	↑	↓ Wnt/β-catenin ↓ Migration ↓ EMT	[117, 125]
miR-613	HCC	↓	↑	↓ Proliferation ↓ Invsion ↓ Poor prognosis	[126]
miR-451	Breast CRC	↓	↑	↑ Chemosensitivity ↓ Proliferation ↓ Migration	[127, 128]
miR-145	Colon	↓	↑	↑ Cell cycle arrest, ↓ Growth, ↓ Metastasis	[31]
miR-214	Hepatocellular	↓	↑	↓ Proliferation, ↑ Apoptosis	[32]
miR-125b	Multiple Myeloma	↓	↑	↑ Apoptosis, ↓ Proliferation, ↓ Drug resistance	[33]
miR-295	Lung	↓	↑	↑ Apoptosis, ↓ Invasiveness	[34]
miR-582	Thyroid	↓	↑	↑ Chemosensitivity, ↓ Tumor growth	[35]

*CRC* colorectal cancer, *EMT* epithelial-mesenchymal transition, *ESCC* esophageal squamous cell carcinoma, *HCC* hepatocellular carcinoma, *NSCLC* non-small cell lung cancer

In summary, microRNAs (miRNAs) are really important for controlling YWHAZ, the gene that produces 14–3-3ζ, especially in many types of cancer. A lot of studies show that some tumor-suppressor miRNAs directly attach to the 3′-UTR of YWHAZ mRNA, which basically lowers how much 14–3-3ζ protein the cell makes. In many cancers, these miRNAs become downregulated, and because of that, 14–3-3ζ levels rise and help the tumor grow by boosting processes like uncontrolled cell division, migration, invasion, and even resistance to treatments. When researchers bring these miRNAs back into the cancer cells, many of these harmful behaviors can be reversed. But if 14–3-3ζ is overexpressed again, it cancels out the effect of the restored miRNAs, showing clearly that 14–3-3ζ is a key player in these pathways [118–126].

Researchers have shown this opposite pattern between miRNA levels and 14–3-3ζ expression using several lab methods, like luciferase reporter assays and Western blotting. These studies also reveal that the miRNA 14–3-3ζ link influences major signaling pathways, such as PI3K/AKT, Wnt/β catenin, and p38MAPK/ERK, which are all really important for things like cancer cell survival, spreading, and how tumors respond to treatments. Consequently, targeting the miRNAs that regulate 14–3-3ζ seems like a promising idea for developing new therapies, especially ones that help overcome drug resistance or slow down aggressive tumor behavior. Table 2 shows the main miRNAs known to interact with 14–3-3ζ in different cancers and why these interactions matter.

General, miRNAs play a key role in controlling how much 14–3-3 protein the cell makes, and this has a big impact on cancer-related processes. These small RNA molecules can change the 14–3-3ζ expression in ways that affect tumor growth, invasion, and even treatment response. Because of this, they might become useful tools for new therapeutic strategies. Learning exactly how these miRNAs regulate 14–3-3ζ could help guide the development of treatments that target these pathways more effectively and potentially improve cancer management.

### CircRNAs

Circular RNAs (circRNAs) also regulate 14–3-3 proteins in cancer, mostly through the competing endogenous RNA (ceRNA) mechanism. In this system, circRNAs act like “sponges” by trapping miRNAs that would normally bind to YWHAZ or other related genes. Most research shows that many circRNAs are increased in cancer cells and that they sponge tumor-suppressor miRNAs, which causes higher expression of 14–3-3ζ (YWHAZ). This increase usually pushes cancer progression forward by promoting metastasis, growth, and resistance to different therapies (Table 3). The circRNA/miRNA/14–3-3 network often activates pathways like PI3K/AKT, and these findings are usually confirmed using dual luciferase assays, rescue tests, and animal models [129].

**Table 3:** CircRNA-14–3-3 interactions in various cancers

miRNA	Cancer	Expression		Key Effects	Ref
		circRNA	YWHAZ		
circ_0069094	Breast	↑	↑	↑ Paclitaxel resistance ↑ Proliferation	[108]
circ_0002496	Breast	↑	↑	↑ Proliferation ↑ PI3K/Akt activation	[129]
hsa_circ_0001546	Ovarian	↓	−	↑ Ferroptosis via Tau aggregation	[130]
circ-VPS13C	Ovarian	↑	↑	↑ Cisplatin resistance ↑ Autophagy	[131]
circAGFG1	Breast	↑	↑	↑ Proliferation ↑ Invasion ↑ Glycolysis	[132]
circCDK17	Cervical	↑	↑	↑ Proliferation ↑ Migration ↑ Glycolysis	[133]
circ_C20orf11	Ovarian	↑	↑	↑ Cisplatin resistance ↑ M2 macrophage polarization	[134]
circNHSL1	Gastric	↑	↑	↑ Migration ↑ Invasion ↑ Glutaminolysis	[135]
circ_0008393	Pancreatic	↑	↑	↑ Metastasis, ↑ Gemcitabine resistance	[36]
circSLC8A1	Renal	↑	↑	↑ Proliferation, ↑ Tumor growth	[37]
circ_0001415	Hepatocellular	↑	↑	↑ Migration, ↑ Invasion	[38]
circ_0068080	Colorectal	↓	−	↓ Apoptosis, ↑ Sensitivity to chemotherapy	[39]
circRNA_104682	Lung	↑	↑	↑ Proliferation, ↓ Anemia	[40]
circ_0016344	Esophageal	↑	↑	↑ Tumor progression, ↑ Cell cycle advancement	[41]
circ_0007214	Thyroid	↑	↑	↑ Invasion, ↑ Metastatic potential	[42]

An interesting exception is hsa_circ_0001546, which does not act through the typical sponge method. Instead it binds directly to 14–3-3 proteins and forms a complex that promotes Tau aggregation and ferroptosis in ovarian cancer [130]. This shows that circRNAs can influence 14–3-3 proteins in more than one direction, making them especially important for study. These complex interactions highlight how circRNAs could possibly be targeted in future treatments to fight therapy resistance and slow down tumor progression. Table 3 summarizes the main circRNA–14–3-3 interactions found in cancer so far.

Circular RNAs (circRNAs) are getting a lot of attention lately in cancer research because they seem to play a big role in controlling 14–3-3 proteins. Most of the time, they work through a ceRNA-type mechanism, where the circRNA basically “sponges up” microRNAs that would normally target genes like YWHAZ. When these microRNAs get absorbed, they can’t do their job, so the amount of 14–3-3ζ (YWHAZ) increases. Higher levels of this protein can push cancer cells to grow faster, spread to other areas, and even become less responsive to common treatments. Many studies have found that circRNAs are often upregulated in different cancers, and they usually trap tumor-suppressor microRNAs, which ends up boosting 14–3-3 activity. This activation also turns on several major cancer pathways, especially PI3K/AKT, making the tumor cells even more aggressive. These results are backed up by experiments such as luciferase reporter tests, rescue assays, and even animal studies, so the evidence is pretty strong [130–135].

There’s also one interesting exception: hsa_circ_0001546. Instead of sponging microRNAs, this circRNA directly attaches to 14–3-3 proteins and forms a complex that causes Tau aggregation and ferroptosis in ovarian cancer [130]. This example shows that circRNAs don’t all act in the same way and can sometimes have totally different functions depending on the context. Overall, these interactions suggest that circRNAs could be useful as new therapeutic targets, especially for tumors that develop resistance to chemotherapy. A lot of circRNAs have been linked to specific types of cancer, where they can increase tumor growth or contribute to drug resistance. This highlights how important they might be for understanding how tumors behave. circRNAs also seem to play broader regulatory roles by interacting with 14–3-3 proteins in general. Because they are stable and circular, they might have bigger and more long-lasting roles in cancer than we currently recognize. More research is still needed, but learning more about how circRNAs interact with 14–3-3 proteins could open up new treatment possibilities and help explain how RNA molecules influence cancer development overall [36–42].

### Long non-coding RNAs

Long non-coding RNAs (lncRNAs) also help regulate the expression of 14–3-3ζ (YWHAZ) in cancer, mostly through the ceRNA mechanism. In this system, lncRNAs bind and block tumor-suppressive microRNAs that would normally reduce YWHAZ levels. Since many lncRNAs are highly expressed in cancer, they end up boosting 14–3-3ζ levels, which activates major cancer pathways like PI3K/AKT and Wnt/β-catenin [136]. This leads to more cancer cell growth, movement, invasion, EMT, and higher resistance to therapies. Even though the ceRNA mechanism is the most common, some lncRNAs regulate 14–3-3ζ differently. For example, certain lncRNAs bind to RNA-binding proteins like HuR, which helps stabilize YWHAZ mRNA instead of affecting microRNAs [137].

There is also an important exception. The lncRNA ASH1 works more like a tumor suppressor by increasing miR-375 levels, which then lowers YWHAZ expression in hepatocellular carcinoma [29]. This shows that not all lncRNAs promote cancer, some actually help block cancer progression by reducing 14–3-3ζ. Clinical correlations between lncRNA expression and patient outcomes suggest their potential as prognostic biomarkers across multiple cancer types. The lncRNA-14–3-3 axis represents a promising therapeutic target for cancer treatment. Table 4 summarizes the key lncRNA-14–3-3 interactions in cancer.

**Table 4:** lncRNA-14–3-3 interactions in cancer

LNCRNA	MIRNA	CANCER	EXPRESSION	KEY EFFECTS	REF
ASH1	miR-375	HCC	↓	↑ Tumor suppressor; downregulates cell proliferation	[43]
FOXD1-AS1	miR-3167	Prostate	↑	↑ Proliferation, ↑ Migration; enhances YWHAZ levels	[44]
SNHG12	HuR	Gastric	↑	↑ Proliferation, ↑ AKT/GSK-3β activation	[45]
MLK7-AS1	miR-375-3p	NSCLC	↑	↑ Migration, ↑ Invasion; promotes tumor aggressiveness	[46]
SNHG12	miR-218-5p	Gastric	↑	↑ Metastasis, ↑ EMT, ↑ β-catenin signaling	[47]
H19	miR-340-3p	Breast	↑	↑ Metastasis, ↑ EMT, activates Wnt/β-catenin	[48]
LUCAT1	miR-134-5p	Gastric	↑	↑ Migration, ↑ Proliferation; upregulates YWHAZ	[49]
SNHG14	miR-206	Cervical	↑	↑ Migration, ↑ Proliferation; promotes YWHAZ levels	[50]
LINC00152	miR-149	Colorectal	↑	↑ Tumor growth, modulates apoptosis pathways	[51]
NEAT1	miR-143	Ovarian	↑	↑ Invasion, ↓ Apoptosis; alters cell cycle regulation	[52]
MALAT1	miR-1	Lung	↑	Promotes EMT, ↑ Cell migration, enhances tumor progression	[53]
TUG1	miR-145	Bladder	↑	↑ Proliferation, ↑ Invasion; involved in metastasis	[54]

Long non-coding RNAs (lncRNAs) regulate the expression of 14–3-3ζ (YWHAZ) in cancer primarily through competing endogenous RNA (ceRNA) mechanisms. In this context, lncRNAs sequester tumor-suppressive microRNAs that target YWHAZ mRNA, leading to increased levels of 14–3-3ζ. Most studied lncRNAs are upregulated in various cancers and contribute to tumor progression by activating key oncogenic pathways such as PI3K/AKT and Wnt/β-catenin. These interactions enhance cancer cell proliferation, migration, invasion, epithelial-mesenchymal transition (EMT), and resistance to therapy. While the ceRNA mechanism is predominant, some lncRNAs directly bind RNA-binding proteins like HuR to stabilize YWHAZ mRNA. An exception is the lncRNA ASH1, which functions as a tumor suppressor by elevating miR-375 expression, consequently reducing YWHAZ levels in hepatocellular carcinoma. Clinical correlations have shown a link between lncRNA expression and patient outcomes, suggesting their potential as prognostic biomarkers across multiple cancer types. The lncRNA-14–3-3 axis is thus a promising therapeutic target for cancer treatment.

lncRNAs add another important layer of control over 14–3-3ζ, since many of them can influence how much of this protein gets made and how it functions in cancer cells. Because lncRNAs are involved in so many cancer-related activities, they might actually shape or modify the tumor-promoting effects that 14–3-3 proteins usually drive. Figuring out exactly how different lncRNAs regulate 14–3-3ζ can help us understand more about tumor biology overall, especially since gene regulation in cancer is extremely complex and involves many overlapping networks. Looking at lncRNAs together with 14–3-3ζ shows why cancer research needs an interdisciplinary approach to really untangle these pathways.

## 14–3-3ζ as a therapeutic target in oncology

High expression of 14–3-3ζ has been repeatedly linked to reduced apoptosis, more frequent tumor relapse, and stronger resistance to chemotherapy in many cancers. Because 14–3-3ζ is so commonly overexpressed and strongly tied to poor treatment results and recurrence, it has become a very promising target for new cancer therapies [138, 139]. Even though this protein is also involved in some non-cancer diseases, attempts to design drugs that directly inhibit 14–3-3ζ are still at a pretty early stage. So far, most experimental approaches rely on siRNAs, antisense oligos, or peptide-based inhibitors [140].

### Small Interfering RNA (siRNA) Approaches

Small interfering RNAs are short, double-stranded molecules (about 21 nucleotides) that can specifically destroy a target mRNA, which then reduces the amount of the protein it codes for. This type of RNA interference is widely used to study gene function, but one big challenge is that siRNAs can sometimes silence genes they weren’t meant to target [141, 142].

In lung cancer models, knocking down 14–3-3ζ using siRNA made the cells much more responsive to cisplatin treatment, both in cultured cells and in mouse experiments [55]. Similar results were seen in other cancer cell lines, where siRNA against 14–3-3ζ increased their sensitivity to stress-induced apoptosis and also reduced tumor growth in xenograft settings [143]. Antisense approaches showed the same trend, leading to lower tumor cell proliferation and better responses to apoptosis-inducing drugs. All of this suggests that siRNA or antisense suppression of 14–3-3ζ could be a real strategy for treating cancer, but delivery problems and off-target effects still need to be solved before it can be used clinically [140, 143]. Research on siRNA targeting of 14–3-3ζ looks especially promising for patients whose tumors show high levels of this protein. But we still need more work to make the delivery safer, improve specificity, and test whether this approach can really work in clinical settings long-term.

### Peptide inhibitors: R18 and difopein

R18 is a non-phosphorylated peptide discovered from a phage display library that binds strongly to all 14–3-3 isoforms and blocks their interactions with partner proteins. To make it stronger and easier to deliver into cells, scientists linked two R18 peptides together using a short linker, creating a dimeric inhibitor called “difopein” [144]. Difopein can trigger apoptosis on its own and also boosts the effects of cisplatin in killing tumor cells [6].

In cells transformed with the BCR-ABL oncogene, difopein caused apoptosis partly by releasing and activating FOXO3a, even though it didn’t directly interfere with BCR-ABL binding or tyrosine kinase activity [145, 146]. Interestingly, cancer cells with imatinib-resistant BCR-ABL mutations were especially sensitive to difopein, particularly when used together with other pathway inhibitors [56]. Similar pro-apoptotic effects were seen in glioma cells treated with difopein or after siRNA-mediated knockdown of 14–3-3 proteins [147–152]. In animal models, difopein slowed tumor growth and caused programmed cell death in tumors in nude mice [57]. Even though R18 and difopein work well as broad 14–3-3 inhibitors, they don’t target specific isoforms like 14–3-3ζ which limits their usefulness for highly precise therapies [153]. Right now there are still no isoform-specific inhibitors available, highlighting a major gap in drug development for this protein family [38].

Therefore 14–3-3ζ is emerging as a strong therapeutic target across many cancers. Even though current tools mainly rely on gene silencing or non-specific peptide blockers, researchers are actively searching for new molecules that can selectively disrupt interactions critical for tumor progression [46]. It is expected that future therapies, possibly small molecule inhibitors or shRNAs,will be better at specifically targeting 14–3-3ζ’s oncogenic functions [154]. Developing these kinds of inhibitors could greatly expand cancer treatment options and also improve our understanding of what 14–3-3ζ really does in both healthy and diseased tissues [155] (Table 5).

**Table 5:** Therapeutic targeting of 14–3-3ζ in cancers

MODALITY	MECHANISM	KEY EFFECTS	LIMITATIONS	REF
siRNA Antisense	↓ YWHAZ, ↓ 14–3-3ζ	↑ Apoptosis, ↑ Chemosensitivity, ↓ Tumor growth	Delivery challenges, Off-target effects, Transient activity	[58]
R18 Peptide	Blocks amphipathic groove	↑ Apoptosis, ↑ Cisplatin sensitivity, Induces cell cycle arrest	Poor stability, Non-selective, Short half-life	[59]
Difopein	Disrupts protein–protein interactions (PPI)	↑ Apoptosis, ↓ Proliferation, Induces tumor regression	Non-selective, Delivery issues, Potential immune response	[60]
shRNA	Sustained YWHAZ knockdown	Durable antitumor effects, ↓ Metastasis	Viral delivery risks, Potential off-target effects	[61]
Small Molecules	ζ-specific PPI inhibition	↑ Apoptosis, ↑ Chemosensitivity, Induces cell cycle arrest	Selectivity challenge, Resistance development	[62]
Antibody Therapeutics	Specific antibody targeting	Induces immune-mediated apoptosis, Enhances chemosensitivity	Possible immune tolerance, Production costs	[63]
Gene Editing (CRISPR)	Direct gene knockout	Long-lasting effects, ↑ Apoptosis in targeted cells	Off-target effects, Ethical considerations	[64]
Nanoparticle Delivery	Targeted delivery of siRNA or drugs	Enhanced delivery efficacy, Reduced systemic toxicity	Complex formulation, Limited biodegradability	[65]
Inhibitors (e.g., small-molecule kinase inhibitors)	Target downstream signaling pathways	↓ Tumor growth, ↑ Apoptosis	Resistance development, Off-target effects	[66]

The peptide R18, originally found using a phage display library, can bind strongly to all 14–3-3 protein isoforms and basically blocks them from interacting with their normal partners. To make R18 work better inside cells, researchers linked two R18 units together with a short linker, creating a dimer called difopein (dimeric fourteen-three-three peptide inhibitor). Difopein showed stronger effects, since it could trigger apoptosis on its own and also make cancer cells more sensitive to cisplatin treatment. In cancer cells expressing the BCR-ABL oncogene, difopein pushed the cells toward apoptosis by re-activating the FOXO3a transcription factor, even though it didn’t directly change the 14–3-3/BCR-ABL interaction or the kinase activity itself. Interestingly, difopein worked especially well in cells carrying imatinib-resistant forms of BCR-ABL, and its effects became even stronger when combined with drugs targeting other signaling pathways. Similar results were seen in glioma cells, where either difopein or siRNA knockdown of 14–3-3 proteins promoted apoptosis in vitro. Animal experiments in nude mice also showed that difopein slowed tumor growth and increased programmed cell death [58–66].

But even with these promising outcomes, neither R18 nor difopein can distinguish between different 14–3-3 isoforms, including 14–3-3ζ, which means we still don’t have isoform-specific inhibitors. This remains a big limitation for designing very targeted cancer therapies. While current strategies depend mostly on gene-silencing tools or broad peptide inhibitors, many groups are now working to identify new molecules that can selectively block specific 14–3-3 interactions that drive tumor progression. Future treatments might include more advanced tools, like small-molecule inhibitors or shRNAs, that can focus specifically on 14–3-3ζ’s cancer-promoting activities. Progress in this area would give us better therapeutic options for cancers that depend on 14–3-3ζ and also improve our understanding of how this protein works in both normal and diseased tissues. Peptide inhibitors such as R18 and difopein still appear to be an interesting direction for blocking 14–3-3ζ activity in cancer. Since they disrupt protein–protein interactions, they might offer meaningful therapeutic benefits if they can be refined further [58–66].

## Clinical perspectives

Even though research on 14–3-3ζ has grown a lot, there are still major gaps in fully understanding what this protein does in cancer [156]. One key issue is that we still don’t have genetically engineered mouse models (either knockouts or overexpression systems) that specifically target 14–3-3ζ, consequently studying its real physiological and oncogenic roles is difficult. Most of the current evidence relies on cell culture work or correlations in patient samples. For 14–3-3ζ to be used reliably as a biomarker in clinics, large and more diverse patient studies are needed, preferably using standardized prospective and retrospective analyses [157]. Another challenge is that labs often use different detection protocols, antibodies, and scoring systems, making it hard in compare results across studies [158, 159].

Therapeutically, targeting 14–3-3ζ is tricky because the protein has no enzymatic activity, plays important roles in normal cells and is hard to inhibit in an isoform specific way. Current inhibitors like difopein are broad and non selective, while RNAi methods still face delivery and stability problems. Because of these issues, some researchers suggest focusing on downstream pathways that 14–3-3ζ regulates such as PI3K/Akt as more immediate alternative [160]. Even with these challenges, the clinical potential of targeting 14–3-3ζ is large. As we learn more about how it shapes cancer progression, it could become the central piece of personalized medicine. Better ways to inhibit 14–3-3ζ might lead to improved treatments for whose tumors are especially resistant to current therapies. Strong collaboration between basic science and clinical research will be essential for bringing these discoveries in the world cancer care.

## Critical analysis of 14–3-3ζ in cancer research: gaps, disparities, and implications

The current literature on 14–3-3ζ provides a prosperity of findings, but the implications for cancer research and clinical practice require deeper investigation. This section aims to critically analyze these findings, emphasizing the comparative insights, discrepancies, and essential gaps in our understanding.

Frequent studies have established the connection between 14–3-3ζ expression and cancer progression across numerous tumor types. For example, high levels of 14–3-3ζ are constantly linked to poor prognostic outcomes, as seen in colorectal cancer and glioblastoma. However, the extent of this correlation can vary considerably between studies. In glioblastoma, some research indicates that raised 14–3-3ζ levels result in shorter survival times, whereas other studies propose that other factors, such as the tumor microenvironment or co-expressing proteins, can moderate these effects. This inconsistency underscores the importance of considering related factors when evaluating 14–3-3ζ as a biomarker.

Although the general consensus is that improved 14–3-3ζ contributes to cancer progress, the mechanisms remain ineffectively considered. For instance, the role of 14–3-3ζ in mediating chemoresistance appears to differ significantly among cancer types. Some studies highlight that targeting 14–3-3ζ can sensitize tumors to chemotherapy, while others present varied results, suggesting that compensatory pathways might activate in response to therapeutic approaches. This difference indicates a substantial gap in our mechanistic understanding of 14–3-3ζ and its downstream effects.

Although the growing body of research, several gaps persist in our understanding of 14–3-3ζ's role in oncology. Limited studies observe the effects of post-translational modifications, which can impact the protein's function and interactions with signaling pathways. Moreover, there is a lack of comprehensive genomic analyses that recognize tumor-specific mutations in 14–3-3ζ across various cancers, which could reveal potential biomarkers for tailored therapies. Addressing these gaps is important for proceeding the utility of 14–3-3ζ in clinical backgrounds. A more nuanced understanding of how this protein interacts within different cancer settings could lead to new therapeutic strategies aimed at moderating its activity. For instance, combining 14–3-3ζ inhibitors with current therapies may enhance treatment efficacy in resistant tumors. Moreover, establishing 14–3-3ζ as a reliable prognostic biomarker could refine patient stratification, allowing for more personalized treatment methods.

In conclusion, while existing studies emphasize the significance of 14–3-3ζ in cancer biology, further critical analysis is essential to delineate the complexities of its role. By linking these gaps and synthesizing current knowledge with clinical implications, we can better inform future research efforts aimed at improving patient outcomes in oncology.

## Conclusions

High levels of 14–3-3ζ are consistently associated with poorer outcomes in many cancers, such as more metastasis, stronger resistance to therapy, and a higher risk of relapse. It behaves almost like an oncogenic hub, helping tumor cells stay alive, grow without attachment, and cooperate with key drivers like HER2. Because of this, 14–3-3ζ is turning into a useful biomarker for picking out high-risk patients who might need more intensive or different treatments in clinical settings. Experimental studies also show that reducing 14–3-3ζ expression can slow tumor growth and make cancer cells easier to kill with chemotherapy. Although further clinical validation is still needed, 14–3-3ζ clearly stands out as a powerful prognostic marker and a promising drug target for overcoming treatment resistance in cancer. Overall, 14–3-3ζ is becoming a central topic in cancer research because of its strong role in tumor progression and therapy resistance, offering new chances for precision medicine in the future for patients. Better understanding how 14–3-3ζ works in cancer will help confirm its clinical value and guide the development of treatments tailored to each patient’s tumor biology, which could significantly improve care in the future.

## Data Availability

Data sharing not applicable to this article as no datasets were generated or analyzed during the current study.
